# *RET* Proto-Oncogene Mutational Analysis in 45 Iranian Patients Affected with Medullary Thyroid Carcinoma: Report of a New Variant

**DOI:** 10.1155/2021/7250870

**Published:** 2021-11-03

**Authors:** Elia Damavandi, Fatemeh Vand-Rajabpour, Maliheh Javadi-Arjmand, Mohammad-Reza Mohajeri Tehrani, Bagher Larijani, Majid Kabuli, Mohsen Ghadami

**Affiliations:** ^1^Specialized Medical Genetic Center (SMGC) of ACECR, Tehran, Iran; ^2^Department of Photo Healing and Regeneration, Medical Laser Research Center, Yara Institute, ACECR, Tehran, Iran; ^3^Department of Medical Genetics, Faculty of Medicine, Tehran University of Medical Sciences, Tehran, Iran; ^4^Endocrinology and Metabolism Clinical Sciences Institute, Tehran University of Medical Sciences, Tehran, Iran; ^5^Cardiac Primary Research Center, Tehran Heart Center, Tehran University of Medical Sciences, Tehran, Iran

## Abstract

**Background:**

The aim of this study was to identify germline mutation of the *RET* (rearranged during transfection) gene in patients with medullary thyroid carcinoma (MTC) and their first-degree relatives to find presymptomatic carriers for possible prophylactic thyroidectomy. *Methods/Patients*. We examined all six hot spot exons (exons 10, 11, 13, and 14–16) of the *RET* gene by PCR and bidirectional Sanger sequencing in 45 Iranian patients with MTC (either sporadic or familial form) from 7 unrelated kindred and 38 apparently sporadic cases. First-degree relatives of *RET* positive cases were also genotyped for index mutation. Moreover, presymptomatic carriers were referred to the endocrinologist for further clinical management and prophylactic thyroidectomy if needed.

**Results:**

Overall, the genetic status of all of the participants was determined by *RET* mutation screening, including 61 affected individuals, 22 presymptomatic carriers, and 29 genetically healthy subjects. In 37.5% (17 of 45) of the MTC referral index patients, 8 distinct *RET* germline mutations were found, including p.C634R (35.3%), p.M918T (17.6%), p.C634Y (11.8%), p.C634F (5.9%), p.C611Y (5.9%), p.C618R (5.9%), p.C630R (5.9%), p.L790F (5.9%), and one uncertain variant p.V648I (5.9%). Also, we found a novel variant p.H648R in one of our apparently sporadic patients.

**Conclusion:**

*RET* mutation detection is a promising/golden screening test and provides an accurate presymptomatic diagnostic test for at-risk carriers (the siblings and offspring of the patients) to consider prophylactic thyroidectomy. Thus, according to the ATA recommendations, the screening of the *RET* proto-oncogene is indicated for patients with MTC.

## 1. Introduction

Calcitonin-secreting parafollicular C cells of the thyroid gland are the origin of 5–10% of thyroid cancers, so called medullary thyroid carcinoma (MTC). MTC has a worse prognosis than the most common form of thyroid cancer, papillary thyroid carcinoma (PTC), which accounts for 60–80% of thyroid carcinomas and originates from follicular cells [1]. MTC has two forms of sporadic (isolated) and hereditary that compromise about 75% and 25% of the cases, respectively. Hereditary forms are transmitted as autosomal dominant pattern of inheritance and are seen either as isolated familial MTC (FMTC) with a prevalence of 10% or as a syndromic form of cancer, also known as multiple endocrine neoplasia type 2 (MEN2A and MEN2B, which have a prevalence of 85% and 5%, respectively) [2, 3]. In familial medullary thyroid carcinoma, the only lesion present is MTC, but MEN2A is characterized by MTC and pheochromocytoma with or without parathyroid hyperplasia or adenoma [1–3].

PTC and MTC have clear clinical and pathological differences, but *RET* proto-oncogene contributes somehow to the carcinogenesis of both types. Activation of the *RET* gene by rearrangement (inversion or translocation) appears to be only seen in patients suffering from PTC [4] or thyroid adenoma [5], and germline missense mutations of the *RET* gene have been shown to be the cause of the hereditary form of MTC (MEN2) [6]. Interestingly, somatic mutations in *RET* have been found in 23–70% of sporadic MTCs and 10–15% of sporadic pheochromocytoma patients, as well [7, 8]. Gain of function mutations in exons 10, 11, 13, 14, and 15 of the *RET* gene have mostly been reported MEN2A (e.g., codons 609, 611, 618, 620, and 634) but about 95% of the patients with MEN2B have a mutation in exon 16 (codon 918). Therefore, to obtain a reliable screening genetic test for MTC patients, exons 10, 11, 13, 14, 15, and 16 should be considered as the hot spots for *RET* gene mutations.

Identification of the germline mutation of the *RET* gene in a patient with MTC provides an accurate presymptomatic diagnostic test for the siblings and offspring of the patients. Here, we report the results of the mutational analysis of the *RET* proto-oncogene in 45 Iranian patients with MTC (either sporadic or familial form) from 7 unrelated kindreds and 38 apparently sporadic cases.

## 2. Subjects and Methods

### 2.1. Patients and Specimen Collection

Seven unrelated families and 38 apparently sporadic cases with medullary thyroid carcinoma referred to Endocrinology and Metabolism Research Institute of Tehran University of Medical Sciences participated in this study. First, 45 index cases whose MTC was diagnosed by an endocrinologist and confirmed histopathologically and underwent thyroidectomy based on the ATA guideline were genetically consulted. Then, each index case was evaluated for mutations in six hot spot exons of the *RET* gene, and segregation analysis was done for their at-risk relatives if a causative mutation was found [3]. Overall, 112 participants were examined for *RET* mutations. All procedures were in accordance with the ethical standards of Tehran University of Medical Sciences and the Helsinki Declaration of 1975 revised in 1983. Written informed consent was obtained from all participants or their parents for their participation and also their permission for the publication of the results.

### 2.2. Molecular Analysis

Genomic DNA was extracted from EDTA peripheral venous blood using the standard salting-out/proteinase K method. Primers were designed to amplify all six exons, 10, 11, 13, 14, 15, and 16, and their exon-intron boundaries using the NCBI Primer-design tool (https://www.ncbi.nlm.nih.gov/tools/primer-blast/) and Gene Runner software (version 6.0.28). Fifty nanograms of the extracted DNA were used as a PCR template. PCR conditions are available upon request. The PCR products were run on a 2% agarose gel, and subsequently, all six exons were bidirectionally sequenced using the ABI3130 automated sequencer and analyzed with the Chromas version 2 (http://chromas.software.informer.com/2.0/). The sequence data were compared with the *RET* sequence data (Ref Seq NM_020975) from the NCBI BLAST human database (http://www.ncbi.nlm.nih.gov/BLAST/) and Ensembl human database (GRCh37).

### 2.3. In Silico Analysis

A comprehensive in silico analysis was performed to evaluate the pathogenic potential of the detected variants by checking in Ensembl.org, dbSNP (http://www.ncbi.hlm.nih.gov/snp), 1000 Genomes databases (http://browser.1000genome.org), ClinVar, HGMD® Professional, MEN2 database (http://www.arup.utah.edu/database/MEN2/MEN2_display.php), and recently published articles. Additionally, Polyphen 2, SIFT, Mutation Tasterو, and InterVar (http://wintervar.wglab.org/) were investigated to predict the possible pathogenic effect of novel variants.

## 3. Results

### 3.1. Clinical and Molecular Findings

Of 45 referred MTC patients, 7 cases had FMTC or MEN syndrome, with more than one affected members. Thirty-eight patients seemed to be apparently sporadic MTC. All six *RET* exons (10, 11, 13, 14, 15, and 16 exons) were sequenced in 45 index patients. Eight *RET* known distinct mutations, one unclassified variant, and one novel variant were detected (Table 1).

**Figure 1:**
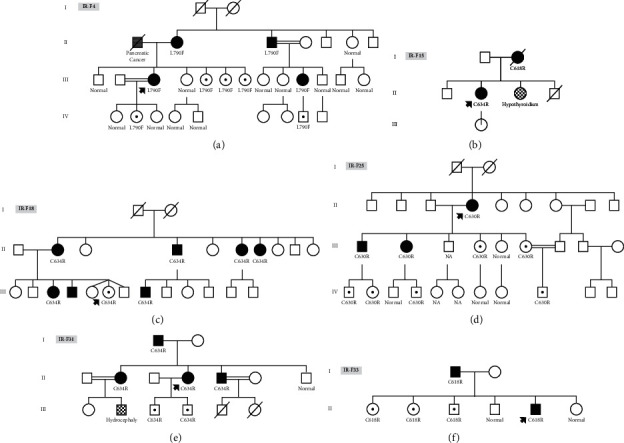
Pedigrees of families with MEN2: (a) IR-F4, (b) IR-F15, (c) IR-F18, (d) IR-F25, (e) IR-F31, and (f) IR-F33. Blackened symbols: affected individuals; dotted symbols: asymptomatic carriers; other symbols: individuals with symptoms other than MEN2; and dashed symbols: deceased.

The family IR-F4 was an extended kindred with two affected individuals in the second generation (II_2_ and II_3_), two affected (III_3_ and III_10_), three carriers in the third generation (III_5–7_), and two carriers in the fourth generation (IV_2_ and IV_8_) (Figure 1(a)). The 44-year-old proband III_3_ and other affected individuals of the pedigree had a known heterozygous mutation (c.2370G > T) in exon 13 (p.L790F). According to the ATA risk classification, p.L790F mutation is stratified as ATA-MOD (A level), indicating a moderate risk of aggressive MTC with ∼10% incidence of PHEO. Carriers of this mutation are advised to undergo prophylactic thyroidectomy only if it occurs as FMTC, or they have a high level of calcitonin. Therefore, these carriers were referred to the endocrinologist for more clinical follow-up.

The proband of the family IR-F15 (II_2_) also had a known mutation (c.1900T > C) in exon 11 (p.C634R). Her mother (I_2_) died because of MTC and was not available but two other family members did not have any *RET* mutations in the six tested exons (Figure 1(b)).

The family IR-F18 was another family with three affected daughters (II_2_, II_6_, and II_7_), one affected son (II_4_), three affected grandchildren (III_3_, III_4_, and III_8_), and one carrier granddaughter (III_6_) (Figure 1(c)). A known heterozygous mutation in exon 11 (c.1900T > C (p.C634R)) was found in all affected individuals (II_2_, II_4_, II_6_, II_7_, III_3_, III_4_, and III_8_) and one carrier granddaughter (III_6_). Interestingly, individual III_6_ had two genetically normal triplets. According to the ATA risk classification, she was advised to undergo prophylactic thyroidectomy before 5 years of age.

The family IR-F25 was an extended kindred consisting of a healthy father (II_3_) and an affected mother (II_4_) with one affected son (III_1_), one affected daughter (III_2_), two carrier daughters (III_4_ and III_6_), one phenotypically healthy son (III_3_), one genetically healthy daughter (III_5_), three carrier grandsons (IV_1_, IV_4_, and IV_9_), one carrier granddaughter (IV_2_), and three genetically healthy grandchildren (IV_3_, IV_7_, and IV_8_) (Figure 1(d)). The 63-year-old proband (II_4_) was diagnosed with MEN2A (MTC, pheochromocytoma (PHEO), and hyperparathyroidism) and underwent thyroidectomy, parathyroidectomy, and adrenalectomy. Sequencing of six *RET* exons of the proband and her affected children revealed a heterozygous mutation (c.1888T > C) in exon 11 (p.C630R) classified as a pathogenic variant. All carrier relatives of c.1888T > C mutation with elevated calcitonin levels, III_4_, III_6_, IV_1_, IV_4_, and IV_9_, underwent prophylactic thyroidectomy at the time of diagnosis (age: 36, 24, 13, 12, and 5 years old, respectively). The 19-year-old carrier granddaughter (IV_2_), who had a normal calcitonin level, did not undergo prophylactic thyroidectomy. However, according to the ATA risk level, p.C630R *RET* mutation is stratified as ATA-MOD (level B) with a moderate risk of aggressive MTC, and the patients are recommended to receive prophylactic thyroidectomy before the age of 5 years old.

The family IR-F31 was another kindred comprising an affected father (I_1_), a healthy mother (I_2_), two affected daughters (II_2_ and II_4_), one affected son (II_5_), one genetically healthy son (II_7_), and two carrier grandsons (III_3_ and III_4_) (Figure 1(e)). MEN2A was diagnosed in the 45-year-old grandfather as the proband with clinical manifestations of MTC and pheochromocytoma. He underwent thyroidectomy and adrenalectomy. His affected children also had MEN2A, including MTC and pheochromocytoma. We identified a heterozygous known mutation in exon 11 (c.1900T > C, p.C634R) in this family which has been classified as a pathogenic variant with a high ATA risk (ATA-H, level C). Prophylactic thyroidectomy is recommended for this mutation before 5 years of age because of the high risk of aggressive MTC. To conduct a follow-up study, two carrier grandsons (III_3_ and III_4_) were referred to the endocrinologist and underwent prophylactic thyroidectomy at age 5.

The family IR-F33 had a known *RET* mutation (c.1852T > C) in exon 10 (p.C618R) (Figure 1(f)). The proband (II_5_) was first referred for *RET* genetic testing because of a high calcitonin level (above 1,500), and histopathological results showed MTC. Initially, a heterozygous known mutation (c.1852T > C) was seen in exon 10 (p.C618R) in the proband (II_5_) at age 27, and subsequently, this variant was found in his affected father I_1_ and three carrier siblings (II_1_, II_2_, and II_3_). According to the ATA risk classification, p.C618R mutation is stratified as ATA-MOD (level B), and carriers are recommended to undergo prophylactic thyroidectomy before 5 years of age. Follow-up visits showed that the calcitonin level was above the normal range (300 pg/ml) in II_5_ even after prophylactic thyroidectomy.

Thirty-eight index patients who seemed to be apparently sporadic MTC were tested for *RET* mutation at six mentioned exons. The results showed that 10 out of 38 patients had germline *RET* mutations. In addition, in four families, we found four carriers of a causative mutation in presymptomatic children who had affected parents, including family IR-F14 with c.1832G > A mutation in exon 10 (p.C611Y), families IR-F17 and IR-F29 with c.1900T > C mutation in exon 11 (p.C634R), and family IR-F22 with c.1901G > T mutation in exon 11 (p.C634F).

In the family IR-F17, a 32-year-old affected mother (II_8_) with a p.C634R mutation had a 4-year-old daughter with elevated calcitonin who was found to have the same mutation as observed in her mother. The proband in the family IR-F29 (III_16_) had an asymptomatic 10-year-old boy who carried the same mutation (p.C634R). Moreover, a 15-year-old carrier of p.C611Y in the family IR-F14 with a moderate ATA (ATA-MOD, Level B) and a 12-year-old carrier of p.C634F in the family IR-F22 were found. All of the carriers in these four families were referred to the endocrinologist for further clinical follow-up and prophylactic thyroidectomy. Furthermore, three index patients in families IR-F11, IR-F23, and IR-F37 had a known *RET* germline mutation (c.2753T > C) in exon 16 (p.M918T), which is a MEN2B specific mutation with the highest ATA risk (ATA-HST, level D). However, no available persons in their families had this mutation. The apparent sporadic patient IR-F39 had a known *RET* mutation (c.1901G > A) in exon 11 (p.C634Y). Likewise, another apparently sporadic patient (IR-F38) had a *RET* germline mutation (p.C634Y) and a novel uncertain variant (c.1973A > G) causing p.H658R, both in exon 11. *RET* novel variant p.H658R was predicted to be of uncertain significance by InterVar, benign by polyphen2, disease causing by mutation taster, and tolerated with SIFT score 0.5 by SIFT web server (Table 2). *RET* sequencing results of another sporadic patient (IR-F19-II_7_) showed an uncertain variant, c.1942G > A, in exon 11 (p.V648I). This variant was predicted to be likely benign by InterVar, benign by polyphen2, disease causing by mutation taster, and tolerated with SIFT score 0.43 by SIFT web server (Table 2).

## 4. Discussion

**Figure 2:**
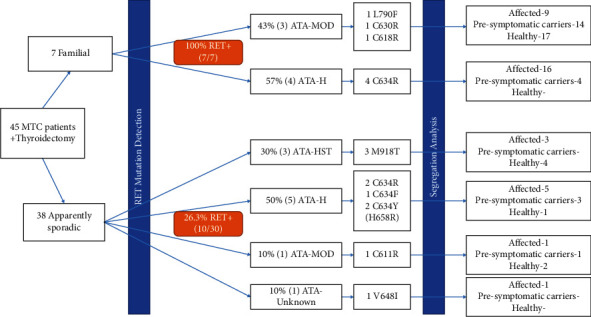
Flow diagram of RET mutations and the ATA risks for 45 Iranian patients affected with medullary thyroid carcinoma.

We examined all six exons (10, 11, 13, 14, 15, and 16) by PCR and direct Sanger sequencing. First-degree relatives of *RET* positive cases were also genotyped and underwent prophylactic thyroidectomy if they had the causative mutation. According to the results (Figure 2), 37.5% (17 of 45) of the MTC referral index patients were found to have eight distinct *RET* germline mutations, including p.C634R (35.3%), p.M918T (17.6%), p.C634Y (11.8%), p.C634F (5.9%), p.C611Y (5.9%), p.C618R (5.9%), p.C630R (5.9%), p.L790F (5.9%), and an uncertain variant p.V648I (5.9%). In addition, we found one new variant in an apparently sporadic patient. The distribution of the detected *RET* mutations was as follows: 64.7% in exon 11, 17.6% in exon 16, 11.8% in exon 10, 5.9% in exon 13, and no mutation in exons 14 and 15 was found. Overall, the genetic status of all participants (61 affected individuals, 22 presymptomatic carriers, and 29 genetically healthy) was determined by *RET* mutation screening, regarding all six exons of RET (10, 11, 13, 14, 15, and 16). Moreover, 22 genetically presymptomatic carriers were referred to the endocrinologist for further clinical management.

Medullary thyroid cancer (MTC) is responsible for about 10% of thyroid cancers, but its prognosis is worse than the most common thyroid cancer, papillary thyroid cancer (PTC). However, 25% of MTCs are hereditary, known as MEN2 syndromes, including MEN2A and MEN2B. Germline missense mutations in hot spot exons of the *RET* gene (exons 10, 11, 13–16) cause 90% of all MEN2 syndromes. Biochemical tests used for screening at-risk cases of MEN2 have false positive/negative results. By contrast, *RET* mutation detection is a promising/golden screening test. Accurate identification of genotype-phenotype correlation in MEN2 syndromes is essential for presymptomatic detection of carriers and clinical management of the affected subjects. In addition, *RET* genetic testing can contribute to distinguishing between three types of MEN2 syndromes that have some manifestation similarities.

According to the results, 37.5% (17 out of 45) of the MTC index patients had nine distinct *RET* germline mutations, including p.C634R (35.3% in 6 patients), p.M918T (17.6% in 3 patients), p.C634Y (11.8% in 2 patients), p.C634F (5.9% in 1 patient), p.C611Y (5.9% in 1 patient), p.C618R (5.9%-in 1 patient), p.C630R (5.9%-in 1 patient), and p.L790F (5.9%-in 1 patient). The distribution of the detected mutations in hot spot exons of *RET* gene was as follows: 64.7% (11/17) in exon 11, 17.6% (3/17) in exon 16, 11.8% (2/17) in exon 10, 5.9% (1/17) in exon 13, and no mutations in exons 14 and 15 were found. We were able to determine the clinical condition of all 112 participants by *RET* screening test, including 61 affected individuals, 22 presymptomatic carriers, and 29 genetically healthy subjects. Presymptomatic carriers were referred to the endocrinologist for further clinical evaluation and possible prophylactic thyroidectomy.

In this study, 7 Iranian kindred with hereditary MTC had four distinct *RET* germline mutations (4 families had p.C634R mutation, and three other families had p.C630R, p.L790F, and p.C618R mutations). Three families (IR-F18, IR-F31, and IR-F36) with p.C634R mutation had FMTC or other MEN2A types phenotypes (MTC, PHEO, and PHPT). Based on the ATA guideline, p.C634R mutation is seen in MEN2A syndromes. Our findings support the role of p.C634R mutation in MEN2A manifestation. The ATA guideline also indicates that p.C634R mutation is seen in FMTC (as a type of MEN2A). Substitution mutations at codon 634 in exon 11, especially cytosine to arginine, are predominately seen in Iranian MEN families [10–14]. It has been stated that mutations at codon 634 are the most frequent mutation in Caucasians [15]. This mutation comprised 53% of all detected mutations in this study. The present study showed that mutations in exon 11, especially at codon 634, are commonly observed in the Iranian population, which is consistent with other studies [10–14].

Interestingly, follow-up studies in the IR-F25 family showed that a 19-year-old carrier, IV-2, did not manifest any MEN2A phenotypes, unlike other carriers. The carrier IV-2 might have some other modifier genes that give her an opportunity to be a healthy carrier with a normal calcitonin level until 19 years of age. In addition, a review of the literature showed that p.L790F mutation is reported for the second time in Iranian families with FMTC [16]. Similarly, it is the second report of p.C618R mutation in Iranian MTC families [13]. Nevertheless, p.C618R mutation is the most common mutation in MEN2A in Saudi Arabian families [17].

The present study showed five distinct *RET* mutations in ten out of thirty-eight (26.3%) apparently sporadic Iranian MTC cases, including two p.C634R, two p.C634Y, one p.C634F, three p.M918T, and one p.C611Y mutations. Additionally, one *RET* uncertain variant (p.V648I) was detected in one apparently sporadic MTC Iranian patient for the first time. Moreover, it is the first report of an apparently sporadic MTC Iranian patient who had a p.C634R mutation and a novel H658R uncertain variant. Further molecular studies are needed to identify the clinical effect of these two variants.

On the other hand, in this study, p.M918T mutation was another common mutation in sporadic Iranian MTC cases, which is inconsistent with findings in European populations [11, 16]. To the best of our knowledge, p.M918T mutation has been reported in two cases and has not been detected in Iranian Azari and other populations [10–14]. Different genetic backgrounds could be the reason for the mentioned conflicting results, which requires more investigations. On the other hand, it seems that some modifier polymorphisms that were found in some carriers of *RET* mutation in our study might contribute to MTC manifestations [18]. Additional genetic evidence is required to clarify the additive effect of modifier polymorphisms.

In conclusion, evaluation of *RET* mutations in hot spot exons resulted in the genetic diagnosis of 37.5% of the Iranian MTC patients. The genotype of all participants in this study was precisely determined, and 22 presymptomatic carriers were referred for further clinical management and prophylactic thyroidectomy. Thus, *RET* mutation screening could serve as an essential detection method for any MTC patient to find presymptomatic *RET* carriers.

## Figures and Tables

**Table 1:** RET mutations and number of affected subjects, carriers, and healthy individuals.

Family ID	Type	Mutation (polymorphism)	Affected number (percent)	Affected individuals (diagnosed primarily with symptoms)	Presymptomatic carriers (diagnosed primarily with genetic testing)	ATA risk based on 2015 ATA risk guideline (ATA risk based on 2009 ATA risk guideline)
*Familial Cases*						
IR-F4	MEN2A	L790F (Polymorphism: L769L, S836S)	4 (11.4%)	II-2, II-3: Confirmed histopathologically and underwent thyroidectomy Ctn level (before surgery): high Ctn level (after sugery): decreased to normal level	III-5: Ctn level: high Thyroidectomy done	MOD (A)
					III-6: Ctn level: high Thyroidectomy done	
				III-3: Ctn level (before surgery): high Pathologic staging: T1 N0 MX Ctn level (after sugery): decreased to normal level	III-7: Ctn level: high Thyroidectomy done	
				III-10: Ctn level (before surgery): high Pathologic staging: T1 NX MX Ctn level (after sugery): decreased to normal level	IV-2: Prophylactic thyroidectomy recommended	
					IV-8: Prophylactic thyroidectomy recommended	
IR-F15	MTC	C634R	2 (5.7%)	I-2: Died		H (C)
				II-2: Ctn level: 320 pg/mL Ctn level (after sugery): decreased to normal level		
IR-F18	MEN2A	C634R (Polymorphism: G691S, S904S)	8 (22.8%)	II-2, II-4, II-6, II-7, III-3, III-4 and III-8: Confirmed histopathologically. Thyroidectomy done Ctn level (before surgery): high Ctn level (after sugery): decreased to normal level	III-6: Thyroidectomy recommended	H (C)
IR-F25	MEN2A	C630R (Polymorphism: G691S, L769L, S904S)	3 (8.6%)	II-4: Thyroidectomy, parathyroidectomy and adrenalectomy done. Ctn level (before surgery): high Ctn level (after surgery): 1.67 pg/mL	III-4: Ctn level: >1000 pg/mL Prophylactic thyroidectomy done.	MOD (B)
					III-6: Ctn level: 62 pg/mL, Prophylactic thyroidectomy done.	
				III-1: Thyroidectomy done. Ctn level (before surgery): high Ctn level (after sugery): 11.7 pg/mL	IV-1: Ctn level: high, Prophylactic thyroidectomy done.	
					IV-2: Ctn level: Normal Prophylactic thyroidectomy recommended	
				III-2: Thyroidectomy, parathyroid and adrenalectomy done. Ctn level (before surgery): >1000 pg/mL Ctn level (after surgery): NA	IV-4: Ctn level: high, Prophylactic thyroidectomy done.	
					IV-9: Ctn level: high, Prophylactic thyroidectomy done.	
IR-F31	MEN2A	C634R	4 (11.4%)	I-1: Thyroidectomy and adrenalectomy done. (Ctn level: NA)	III-3: Prophylactic thyroidectomy recommended.	H (C)
					III-4: Prophylactic thyroidectomy recommended.	
				II-2: Thyroidectomy, adrenalectomy done. (Ctn level: NA)		
				II-4: Thyroidectomy, adrenalectomy done. (Ctn level: NA)		
				II-5 Thyroidectomy, adrenalectomy done. (Ctn level: NA)		
IR-F33	MEN2A	C618R (Polymorphism: G691S)	2 (5.7%)	I-1: Thyroidectomy done. Ctn level (before surgery): 200 pg/mL Ctn level (after surgery): 20 pg/mL	II-1: Thyroidectomy done. (Ctn: decreased to normal level)	MOD (B)
					II-2: Thyroidectomy done. (Ctn: decreased to normal level)	
				II-5: Thyroidectomy and adrenalectomy done. Ctn level (before surgery): >1000 pg/mL Ctn level (after surgery): 300 pg/mL	II-3: Thyroidectomy done. (Ctn: decreased to normal level)	
IR-F36	MEN2A	C634R	2 (5.7%)	II-2: Thyroidectomy, adrenalectomy done. III-1: Died	III-2, III-4: Prophylactic thyroidectomy recommended.	H (C)

*Apparently Sporadic Cases*						
IR-F11	Metastatic MTC	M918T	1 (2.8%)	Ctn level (before surgery): 8600 pg/mL Thyroidectomy done. Relapse with high Ctn level: 2000 pg/mL. Metastatic MTC reported in histopathology report and sonography of neck		HST (D)
IR-F14	MTC	C611Y (Polymorphism: G691S, L769L)	1 (2.8%)	Confirmed histopathologically and underwent thyroidectomy Ctn level (before surgery): high Ctn level (after sugery): decreased to normal level	III-2: Prophylactic thyroidectomy recommended.	MOD (B)
IR-F17	MEN2A	C634R	1 (2.8%)	Thyroidectomy and unilateral adrenalectomy done. Ctn level (before surgery): 352 pg/mL Relapse with high Ctn level.	III-1: Ctn level: 20 pg/mL Thyroidectomy recommended	H (C)
IR-F19	MTC	V648I (uncertain)	1 (2.8%)	Thyroidectomy done years ago. Relapse with Ctn level of 31 pg/ml.	—	—
IR-F22	MTC	C634F (Polymorphism: G691S, L769L, S836S, S904S + A > T IVS16)	1 (2.8%)	Confirmed histopathologically. Thyroidectomy done. Ctn level (before surgery): 553 pg/mL Ctn level (after surgery): 1.2 pg/mL	III-1: Ctn level: 13.4 pg/mL Thyroidectomy recommended	H (C)
IR-F23	MEN2B (metastatic MTC + ambigous genitalia)	M918T	1 (2.8%)	Metastatic MTC to lymph nodes and both lungs confirmed histopathologically Total thyroidectomy and LND done. Ctn level: >2000 pg/mL	—	HST (D)
IR-F29	MEN2A	C634R	1 (2.8%)	Thyroidectomy and bilateral adrenalectomy done. Ctn level: NA	IV-1: Ctn level: Normal Prophylactic thyroidectomy recommended	H (C)
IR-F37	MTC	M918T	1 (2.8%)	NA	—	HST (D)
IR-F38	MTC	C634Y, H658R (novel)	1 (2.8%)	NA	—	H (C)
IR-F39	MTC	C634Y (Polymorphism: G691S)	1 (2.8%)	NA	—	H (C)

**Table 2:** Variants according to *in silico* analysis.

	Variant	InterVar	PolyPhen2	Mutation taster	SIFT	MEN2 database	Report
1	p.H658R	Uncertain significance	Benign with 0.006 score:Sensitivity 0.97Specificity 0.75	Disease causing	Tolerated (SIFT score 0.5)		Novel
2	p.V648I	Likely benign	Benign with 0.006 score:Sensitivity 0.97Specificity 0.75	Disease causing (NMD, amino acid sequence change, protein features might be affected, and splice site changes)	Tolerated (SIFT score 0.43)	Uncertain	[9] (2002)

## Data Availability

The data used to support the findings of this study are included within the article.
